# State of Charge Estimation of Battery Based on Neural Networks and Adaptive Strategies with Correntropy

**DOI:** 10.3390/s22031179

**Published:** 2022-02-04

**Authors:** Rômulo Navega Vieira, Juan Moises Mauricio Villanueva, Thommas Kevin Sales Flores, Euler Cássio Tavares de Macêdo

**Affiliations:** Renewable and Alternatives Energies Center (CEAR), Electrical Engineering Department (DEE), Campus I, Federal University of Paraiba (UFPB), Joao Pessoa 58051-900, Brazil; jmauricio@cear.ufpb.br (J.M.M.V.); thommas.flores@cear.ufpb.br (T.K.S.F.); euler@cear.ufpb.br (E.C.T.d.M.)

**Keywords:** estimation, state of charge, batteries, correntropy, cost function, Artificial Neural Networks

## Abstract

Nowadays, electric vehicles have gained great popularity due to their performance and efficiency. Investment in the development of this new technology is justified by increased consciousness of the environmental impacts caused by combustion vehicles such as greenhouse gas emissions, which have contributed to global warming as well as the depletion of non-oil renewable energy source. The lithium-ion battery is an appropriate choice for electric vehicles (EVs) due to its promising features of high voltage, high energy density, low self-discharge, and long life cycles. In this context, State of Charge (SoC) is one of the vital parameters of the battery management system (BMS). Nevertheless, because the discharge and charging of battery cells requires complicated chemical operations, it is therefore hard to determine the state of charge of the battery cell. This paper analyses the application of Artificial Neural Networks (ANNs) in the estimation of the SoC of lithium batteries using the NASA’s research center dataset. Normally, the learning of these networks is performed by some method based on a gradient, having the mean squared error as a cost function. This paper evaluates the substitution of this traditional function by a measure of similarity of the Information Theory, called the Maximum Correntropy Criterion (MCC). This measure of similarity allows statistical moments of a higher order to be considered during the training process. For this reason, it becomes more appropriate for non-Gaussian error distributions and makes training less sensitive to the presence of outliers. However, this can only be achieved by properly adjusting the width of the Gaussian kernel of the correntropy. The proper tuning of this parameter is done using adaptive strategies and genetic algorithms. The proposed identification model was developed using information for training and validation, using a dataset made available in a online repository maintained by NASA’s research center. The obtained results demonstrate that the use of correntropy, as a cost function in the error backpropagation algorithm, makes the identification procedure using ANN networks more robust when compared to the traditional Mean Squared Error.

## 1. Introduction

With the development of electric vehicles, the technologies related to energy management systems have been of extreme importance in recent years. One of the main problems is how to control the process of charging and discharging the battery as well as how to extend its useful life [1]. Lithium-ion batteries, in this context, have been intensely used in various electric vehicle and renewable energy applications due to their power density and high energy density, which provides a smaller package volume when compared to other chemical materials in the construction, as well as intrinsic characteristics associated with safety, accelerated charging, and longer operational life [2]. In electric vehicles (EVs) or hybrids (HEVs), the battery pack is one of the most essential elements, and because it is composed of several coupled batteries, there are devices dedicated to monitoring these components: the Battery Management System (BMS) and the Intelligent Battery Sensor (IBS). Both are responsible for collecting data such as voltage, operating current, and temperature and, from this, estimating performance and quality indicators such as State of Charge (SoC), State of Health (SoH), and State of Function (SoF). Among these performance parameters, the SoC and SoH are the variables that stand out the most, because their estimations are more complex and their methods require more robust mechanisms to produce an accurate estimation. The main adversity found in identifying these parameters is their highly non-linear behavior [3].

The systems identification by means of linear models does not allow for satisfactory approximations, especially when the physical aspects of battery discharge operations are considered. Recent studies on batteries have shown that the discharge rates are non-linear in time, and, furthermore, depend on the residual capacity of the battery [4,5,6], i.e., for different operating conditions there are different lifetimes, so the effective capacity of the battery is not the same for different discharge operations [7].

Understanding the battery aging process is a complex task, as many factors, from environmental conditions to the use of the vehicle, generates different aging effects [8]. Battery degradation is accelerated with factors that include, but are not restricted to: the frequency of cycling, large change in the state of charge, large current magnitudes during charging and discharging, high temperatures and exposure to high temperature [8]. The unknown weight and non-linear behavior of each of these factors makes the precise estimation of the SoC a complicated task [9]. It is often difficult to establish a reasonable and precise mathematical model for the charge and discharge process.

In the current practical scenario, there are two main research guidelines for SoC estimation. The first is the nonlinear filtering method based on the equivalent battery model [10], which is the Kalman filter method and the artificial neural network method [11], respectively. However, both guidelines have their disadvantages, the performance of the model estimated by Kalman filter is extremely dependent on the equivalent circuit adopted, while the performance of the model to neural networks requires a sufficiently large database with a suitable cost function to be able to accurately estimate the output of the network, especially when there is the possibility of the output being contaminated with noise and outliers. If combined with each other or with guidelines from other methods, they may be able to overcome their respective shortcomings.

The objective of this paper is to develop a methodology for estimating the SoC of lithium-ion batteries. In order to overcome the limitations that the estimation model based on neural networks presents, this work proposes the development of an ANN that contains as cost function the Correntropy and a training performed from a database from an online repository maintained by NASA’s research center [12]. However, the successful metric application of Correntropy in various scenarios depends on the proper tuning of the width of its *kernel* (represented by the Greek letter σ). This *kernel* is a new parameter intrinsic to this Correntropy-based estimation methodology.

In this paper, in order to properly determine this parameter and consequently use the Maximum Correntropy Criterion (MCC) method, this parameter is determined in two ways according to the data set, as presented in the sequence:

- From genetic algorithms, when the data set has no presence of noise and outliers;
- From adaptive strategies, when the data set presents added noise and outliers, following the expression of [13].

Among the contributions of this work are:

1. Unlike the classical MSE-based function, which is a criterion related only to the second-order moment of the error, the Maximum Correntropy Criterion (MCC) allows statistical moments of higher orders to be taken into account during the fitting of model parameters, such as skewness and kurtosis. These moments may contain relevant dynamic information that should be inserted into the model structure;
2. With the insertion of MCC, there is the possibility that a set of highly discrepant values (*outliers*) can be disregarded or eliminated, through the appropriate selection of the *kernel* width [14].

## 2. Related Works

SoC of batteries has a direct mapping relationship with their external (static) characteristic parameters, such as the open circuit voltage (OCV), impedance, etc. Thus, by measuring their parameters and then using the method of the Lookup Table, which was built with the relationships 88 between SoC and one or more parameters, we can infer the SoC [15,16,17]. However, it is hard to measure the precise OCV in real-time because the measurement of battery OCV requires cutting off the power and having the battery rest for an extended period. On the other hand, the measurement of battery impedance relies on the measurement device, thus, it cannot be implemented for running EVs.

In another context, when the maximum available capacity of a battery is known and its current can be measured precisely, the ampere-hour integral method can permit the accurate calculation of the variation of the SoC. If we know the initial SoC, we can obtain the accurate SoC. This method works very accurately for batteries because there are no significant side effects during normal operation. However, for the estimation of the SoC by this method, there are two drawbacks that need to be dealt with first. First, the initial SoC must be known. Second, the measurement errors of battery current from random disturbances, such as noise and temperature drift, are inevitable.

One of the solutions traditionally approached to deal with nonlinear ones, for SoC estimation, are those based on adaptive systems, in particular Kalman filter (KF) and its variants [18,19,20]. In this method, the battery is seen as a power system and is described as an equivalent circuit. Based on the error between the voltage output of the model and the measured voltage, the KF adjusts the SoC parameters to change the voltage output of the model, in order to minimize the voltage error. However, its performance is extremely dependent on the accuracy of the equivalent model used, which directly reflects its complexity. In the paper published by [21], Lagrange multiplier technique and sigma point Kalman filter (SPKF) is proposed for the lithium-ion battery model identification and state of charge (SoC) estimation, respectively. This model is based on the construction of the state-space model of an RC equivalent circuit. SPKF uses the unscented transformation to handle the non-linearity in the systems. The Kalman filter is an iterative algorithm that requires the experimental survey of the SOC-OCV to construct the Lookup Table for a constant temperature. However, in the literature, the Lookup Table is constructed of the SoC as a function of OCV and temperature, to compensate for the thermal effects in SOC estimation. In this new proposal, the proposed neural network is built based on the black box model, which does not require the construction of state space models. In this proposal, the ANN learns from battery tests, with different operating conditions.

Methods based on Artificial Intelligence, such as Artificial Neural Networks (ANNs) and Fuzzy Neural Networks (FNNs), have been used in the estimation of the SoC, as well as in the estimation of current and temperature parameters [20,22,23]. These methods can be applied in almost all types of batteries and present an excellent non-linear performance. However, SoC estimation by Artificial Neural Networks is extremely dependent on a data set that is robust enough to represent possible operating conditions. In the case where the operating conditions of the system differ from the training data set, the artificial neural network usually presents relevant errors. Fuzzy logic, on the other hand, is dependent on the architecture built by a human expert in that particular domain. If the architecture does not have a vast knowledge base about the problem domain, the system tends to present errors.

From the literature review, it is observed a growing interest in the development of models for SoC estimation, based on new paradigms and theoretical references, that allow to optimize the models and extract the most amount of information from battery tests. This paper analyses the application of Artificial Neural Networks (ANNs) in the estimation of the State of Charge (SoC) of lithium battery. Normally, the learning of these networks is performed by some method based on the gradient, having the mean squared error as a cost function. This paper evaluates the substitution of this traditional function by a measure of similarity of the Information Theory, called Correntropy. This measure of similarity allows for statistical moments of a higher order to be considered during the training process. For this reason, it becomes more appropriate for non-Gaussian error distributions and makes training less sensitive to the presence of outliers.

## 3. Database—National Aeronautics ans Space Administration (NASA)

The proposed identification model was developed using information for training and validation, using a dataset made available in an online repository maintained by NASA’s research center [12]. The data used for the overall objective are generated in the test table described in [24]. In all, the dataset used for training covers 34 batteries, totaling 2794 tests, distributed in continuous charge and discharge tests, dynamic stress tests, and among other types of tests. The variables of interest in this database revolve around discharge current, terminal voltage, operating temperature, and battery capacity after several discharge cycles.

For the development of the algorithm in this work, the discharge tests present in NASA’s database were used, as they provide a good consistency of data and the amount of tests performed is quite satisfactory for the training process, and in a more detailed study, it also makes it possible to verify the degradation of the battery over time. In general, 93% of the available sample set was used for identification model training, in which the others are subdivided among validation tests. This value was defined empirically, the intention being to separate a large enough training data set to contemplate the most diverse operating conditions. Table 1 shows the electrical specifications of the 18.650 battery.

In the selection of variables for the identification process, the main variables that affect the battery’s behavior, such as current, voltage, and temperature, were considered.

At first, the battery voltage was chosen as input for the identification system because it represents the result of all the intrinsic dynamic processes of the battery. The current was selected because it has a direct relationship with the charge available in the battery. The third variable that will compose the model is the temperature. In [25], SoC is defined as the present charge of the battery proportional to its capacity. It indicates the remaining percentages of the battery of an EV. In [26], it is observed that the maximum capacity increases with temperature. This increase is expected from enhanced electrochemical reduction-oxidation (redox) at the anode and cathode as shown by [27,28]. The increase is significant after 35 ${}^{\circ }$C, and it slows down after 45 ${}^{\circ }$C. This implies that temperature plays a relevant role in SoC estimation, since it can be directly relate to the charge capacity present in the battery of an Electric Vehicle.

**Figure 1:**
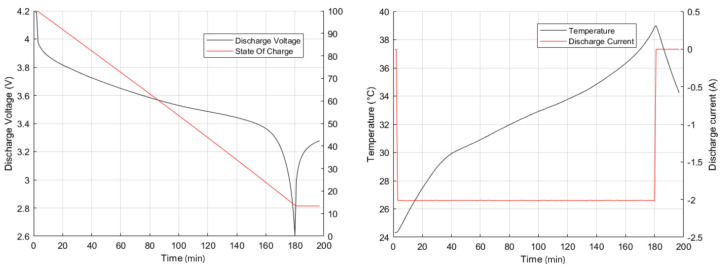
Discharge voltage, current, state of charge and temperature curves in a single test.

Figure 1 shows the variables of interest for a single test. In Figure 1a the operating voltage and the state of charge are observed. The operating voltage stays within the maximum limit (4.2 V) and the breakdown voltage (2.6 V). Figure 1b represents the discharge current of this test (2 A), together with the temperature to which the test was subjected.

A possible limitation for the proposed method is found around the discharge value of the operating current. Although the database is extremely complete in order to provide tests at temperatures considered low (5° to 15 °C), medium (25° to 35 °C) and high (45° to 55 °C), the operating current only has three discharge values, which are: 1 A, 2 A, and 4 A. This restriction of values in the discharge current can cause problems in the estimation of the identification model when, for example, a possible discharge value of 1.5 A is used.

Another limitation of this work is the use of the battery in extreme temperature conditions ($T<0{\;}^{\circ }$C or $T>55{\;}^{\circ }$C). In [29], for example, it is observed that at a temperature of $-10{\;}^{\circ }$C, the average SoC in an electric vehicle is incremented by 3–6% units every hour, because a larger charge current is accepted at the same voltage.

These limitations can be overcome by fine tuning the neural network. The basic idea with fine tuning is to take another very large data set that is hopefully somewhat similar to your domain, train a neural network, and then fine tune this pre-trained network with your more specific data set.

## 4. Background Definitions

In this section, we show the definitions background regarding the Correntropy theory as error minimization criteria for the estimation of the adaptive system parameters.

### 4.1. Adaptive System

Considering that most industrial processes of interest to engineering have a certain degree of inherent nonlinearity, there is an important need to investigate the performance of new techniques derived from artificial intelligence, capable of dealing with nonlinearity in an advanced way.

The systems identification (especially nonlinear systems) using artificial neural networks has been one of the most explored subjects in the model identification literature in the last years (for control purposes or not), with applications in practically all areas of human knowledge that use mathematical models. Specifically, process identification using artificial neural networks began to be explored with the work of [30].

It is believed that the wide use of this technique was only possible due to certain peculiar characteristics of artificial neural networks, such as:

- Potential to model complex dynamics such as those usually presented by nonlinear systems;
- Artificial neural networks can be trained easily (when compared to other techniques), using historical data from the process under study;
- Are easily applied to multivariable systems;
- Have the ability to infer general rules and extract typical patterns from specific examples, and recognize input-output mapping from multi-dimensional complex, multi-dimensional field data.

**Figure 2:**
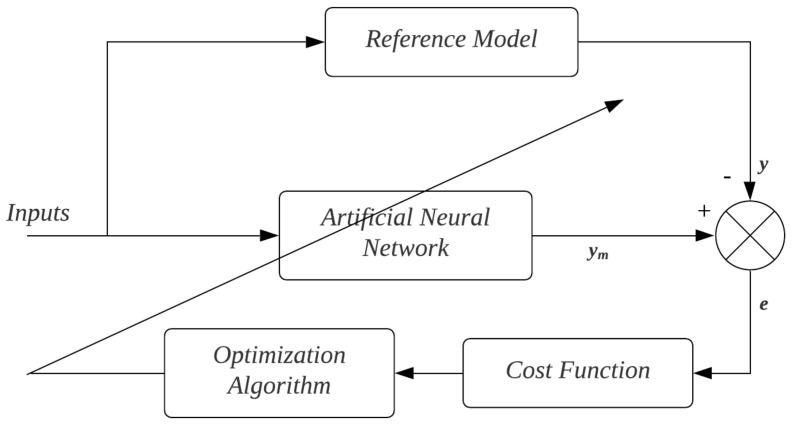
Estimation diagram by reference model.

In this context, in adaptive systems used to process identification, most of the works found in the literature adopt the Mean Squared Error (MSE) as a cost function both for parameter tuning and for performance analysis of the designed models [13], as shown in the diagram of Figure 2. In this Figure, $y_{m}$ is the neural network signal, *y* is the reference model signal, and *e* is the error model signal.

This can be verified by observing that among the works researched so far, which apply ANN to identification tasks, all of them use MSE as a cost function of their learning algorithms. According to [31], the use of MSE in identification problems seems so natural that in most cases this criterion is adopted without questioning. However, as presented by [32], the application of MSE for model parameter fitting is considered optimal if, and only if, the Probability Distribution Function (PDF) of errors presents a Gaussian behavior.

The existence of non-Gaussian distributions has motivated the research for techniques based on minimizing a larger amount of information from the error distribution instead of just minimizing its variance [33]. In this sense, Information Theory provides some measures of similarity, such as entropy and correntropy, which can be used to obtain dynamic models.

Uniting techniques derived from artificial intelligence, such as artificial neural networks, classic methods of identification and the modern theory of control, as well as a more robust cost function, it was shown that these methodologies can be successfully used in the search for better performances of electrical processes under the action of automatic control.

### 4.2. Correntropy Theory

The correntropy is a measure of generalized similarity between two random variables *X* and *Y*, and is mathematically defined by the following expression (1).

(1) $$v_{\sigma }\left(X,Y\right)=E\left[\kappa \left(X,Y\right)\right]=\;\int \int \;\kappa \left(x,y\right)f_{XY}\left(x,y\right)dxdy$$

where: $f_{XY}$ is the joint probability density function of *X* and *Y*, E[.] is the statistical operator and k[.] is a positive symmetric *kernel*.

The concept of correntropy can be applied to the estimation of nonlinear dynamical systems, being used as an evaluation criterion, whose objective is to estimate a model capable of satisfactorily representing the existing dynamic relationships between the input and output variables of the system to be identified [34]. Thus, the main objective is to make, for the same input values, the output of the model obtained during the estimation process, as close as possible to the output of the dynamic system.

The most popular *kernel* used in correntropy is the Gaussian *kernel*, according to Equation (2). Its popularity comes from the fact that it has several advantages. It is universal, and you can integrate it against most functions that you need to.

(2) $$\kappa \left(x,y\right)=K_{\sigma }\left(x_{i}-y_{i}\right)=\frac{1}{\sigma \sqrt{2\pi }}exp\left[-\frac{{\left(x_{i}-y_{i}\right)}^{2}}{2\sigma^{2}}\right]$$

where: σ is the standard deviation, being responsible for defining the width of *kernel*.

The *kernel* width is an adjustable parameter of correntropy that works as a zoom lens capable of controlling the observation window in which the similarity between two random variables is evaluated. The possibility to adjust the size of the *kernel* provides a mechanism that allows very discrepant values outliers in a data set to be disregarded or eliminated [14].

In general, the density and probability function established by Equation (1) is unknown, and usually only a finite amount of data is available. Given this, the correntropy can be calculated by the sample correntropy estimator, which is given by Equation (3) [33].

(3) $$v_{\sigma }\left(X,Y\right)=\frac{1}{N}\sum_{i=1}^{N}\kappa_{\sigma }\left(x_{i},y_{i}\right)$$

where: *N* represents the number of samples available.

### 4.3. Kernel Width—Optimization by Genetic Algorithm and Adaptive Strategy

In general, the training algorithms perform the minimization of some quadratic error function. Among these, the most widely used, regardless of the type of net application, is the Mean Squared Error. In [35], cite some characteristics that justify the popularity of this evaluation function: it has a smooth error surface, it is a continuously differentiable function, has a single global minimum, and its simple computation does not require high computational effort.

Despite its attractive properties, [32,33] state that the mean squared error has optimal application only if the probability density function of the error is Gaussian. In nonlinear problems with high noise, the presence of outliers and non-Gaussian error distribution means the use of this cost function has limitations.

In supervised training algorithms, the comparison between the output provided by the neural net and a desired output value can be interpreted from a statistical perspective. In this case, what you want to analyze is the probability that these two variables are similar [13], making it clear that it is possible to replace the traditional mean square error with some measure of similarity, such as correntropy.

However, the successful application of correntropy depends on the proper choice of the Gaussian *kernel* width. Therefore, this parameter can influence the performance, convergence rate, and robustness of the training algorithm. According to [36], the *kernel* width should be set aiming at a balance between outliers rejection and model estimation efficiency.

In the literature, there can be found some approaches that can be applied to the *kernel* width of the correntropy, such as Silvermann’s rule [37]. Although widely used, this method is not able to provide an optimal value for this parameter. It is usually used to indicate a reference value for further refinement. Silvermann [37] further suggests using an alternative estimator based on interqualite intervals to determine the kernel width, as it is a dispersion method that is more robust to outliers. However, both suggestions work well in practice when the actual density is close to the normal distribution

A possible solution to the *kernel* width optimization problem is the use of genetic algorithms and adaptive strategies. In general, genetic algorithm (GA) has been widely adopted to find quality approximate solutions, even for large scale cases of the problem, in a reasonable time. Therefore, in scenarios where you only want to optimize the *kernel* width, resulting in a reduction of the estimation model error, the genetic algorithm is suitable. Adaptive strategies, on the other hand, are more appropriate when the estimation model is contaminated with noise and outliers, since this strategy allows the estimation model to ignore discrepant measures of the identification process.

## 5. Methodology

The proposed methodology is based on the Maximum Correntropy Criterion (MCC) with Gaussian *kernel* as mechanisms for adaptation and estimation of an Adaptive System by the reference model, which we will call $RNA_{MCC}$, and which is described in the following.

### 5.1. Identification Structure

**Figure 3:**
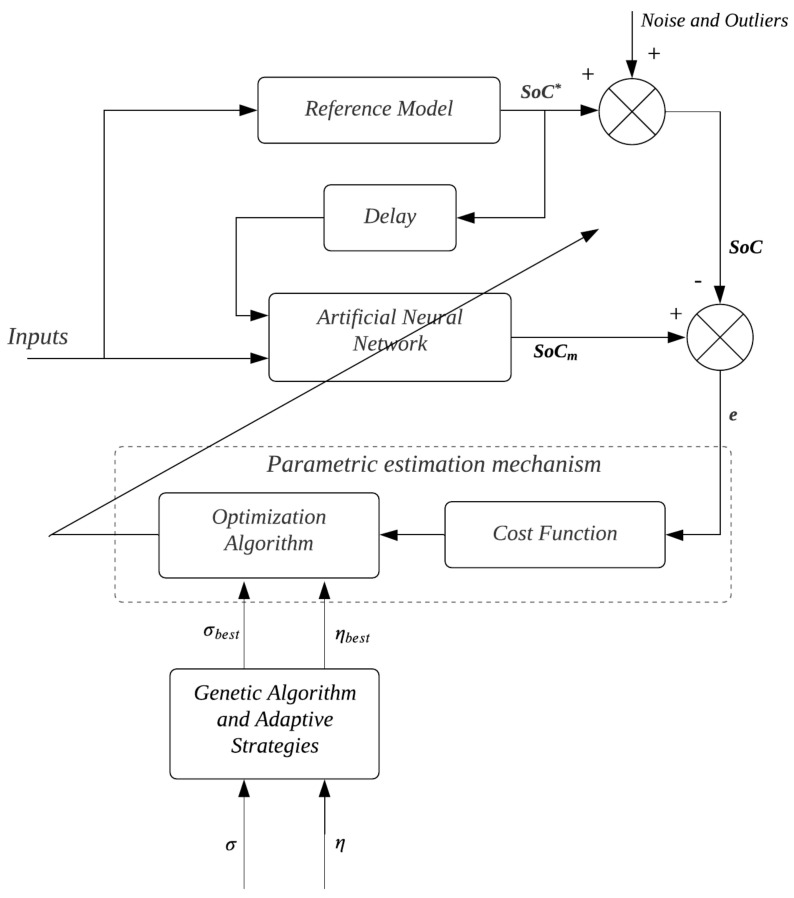
Diagram of the identification structure.

Figure 3 shows the block diagram of the proposed adaptive system by reference model, consisting of five subsystems:

- *Reference Model:* SoC predictor from the Coulomb Counting Method using the NASA online repository database;
- *Artificial Neural Networks*: An adaptive system that allows the maximization of a given cost function in order to minimize the estimation error;
- *Parametric Estimation Mechanism*: Implements Estimation of Neural Networks parameters (Biases and Weights);
- *Genetic Algorithm and Adaptive Strategies*: Algorithm for optimizing kernel width and learning coefficient;
- *Delay*: Allows saving of previous samples of the current value of the state of charge.

In the training algorithm for the state of charge, shown in Figure 3, the *Reference Model* corresponds to SoC estimation by Coulomb Counting. Thus, an epoch is counted at each presentation to the artificial neural network of all input-output pairs present in the training set. Thus, epoch after epoch, the *Parametric estimation algorithm* performs the parameter adjustment of the ANN with the goal of maximizing the correntropy.

As explained earlier, the successful use of correntropy depends on the proper tuning of the *kernel* width and its learning coefficient. Thus, the new parameter plays a decisive role in the performance of the training algorithm. The role of optimizing both parameters in SoC usage scenarios is left to the *Genetic Algorithm and Adaptive Strategies*.

In order to facilitate the reader’s understanding of the next sections, the most important symbols that will be used and their nomenclature have been gathered together, as follows:

- Parameters intrinsic to Correntropy: σ (kernel width) and η (learning coefficient);
- Activation functions of the ANN: V[.] (sigmoid activation function of the intermediate layer) and F[.] (linear activation function of the output layer);
- Parameters intrinsic to ANN: *w* (weights) and *b* (bias);
- Correntropy cost function: ϵ;
- Number of samples: *N*;
- Error per sample between the desired output and the estimated output: $e_{k}$;
- Number of neurons of the output layer: *d*;
- Number of neurons of the hidden layer: *j*;
- Number of inputs: *i*.

### 5.2. Artificial Neural Network

The NARX network is applied as a tool in this work because it is a dynamic and recurrent network with high performance. Its gradient descent learning tends to become more effective due to its built-in memory, which provides a shorter path for propagating the gradient information when the network is open, rather than backpropagating the error signal, giving the network fast convergence and robust performance [38].

**Figure 4:**
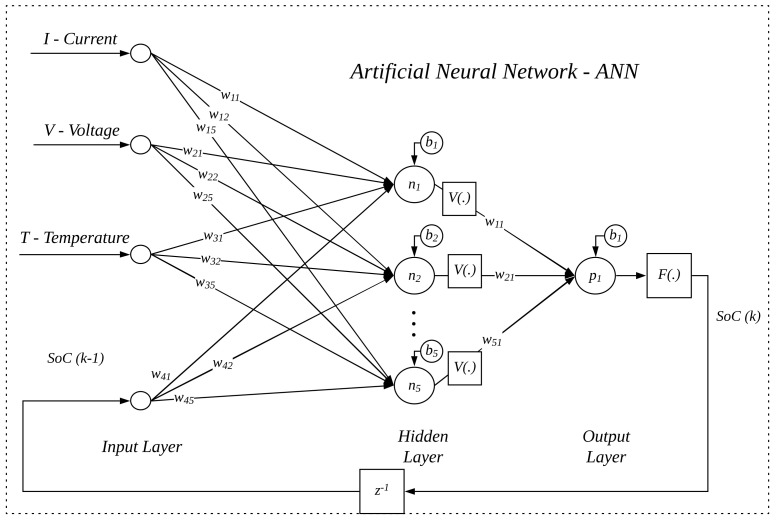
Artificial Neural Network.

The ANN applied in this work, shown in Figure 4, has as its main characteristic the feedback with values obtained at the network’s output, which are added to inputs external to the network. In this way, the output is a function of *k* samples of current inputs together with past outputs. In this figure, *n* represents the number of neurons in the intermediate layer, *p* the number of neurons in the output layer, V[.] is the activation function of the intermediate layer, and F[.] is the linear output activation function of the output layer. Thus, the estimation of SoC(k) is written as a function of the parameters described in Equation (4).

(4) SoC(k)=f(SoC(k−1),I(k),V(k),T(k))

where: I(K) represents the operating current, V(k) represents the discharge voltage, T(k) is the temperature, and SoC(k−1) is the state of charge at the previous sample.

To obtain the network with the highest performance, several tests were performed with different parameters to test the performance of the recursive artificial neural network and determine the choice of the best parameters empirically. The metrics of the identification system analyzed to achieve high performance are: Mean Squared Error (MSE) and Mean Absolute Percentage Error (MAPE). The characteristics of the ANN, represented by the Figure 4 are: (a) single hidden layer; (b) sigmoid activation function for the hidden layer, represented by V(.); (c) linear activation function for output layer, represented by F(.); (d) current inputs (Current, Voltage, and SoC) and past inputs (SoC from the previous samples); and (e) five neurons in the hidden layer. Initially, to define the number of neurons present in the layers, a weighting was carried out. The number of neurons should be large enough to model complex data, but not too large, which could cause the net to specialize too much in training data, losing its generalization power (overfitting).

### 5.3. Parametric Estimation Mechanism

ANNs training is applied to minimize an established cost function and consequently approximate the estimated SoC response by the neural system to the SoC reference, based on NASA’s available data set. Another way is to see that the parameters of an artificial neural network are adjusted by means of a training algorithm that aims to minimize a certain cost function.

According to [33], an identification model can be interpreted as an unknown function f(X,θ), which for a set of *N* input samples X = $x_{1},x_{2},...,x_{N}$ and a vector of *n* adjustable parameters $\theta_{m}$ = ${\left[\theta_{1},\theta_{2},...,\theta_{n}\right]}^{T}$, provides a set of *N* estimated outputs $Y_{m}$ = $Y_{1},Y_{2},...,Y_{N}$. These parameters $\theta_{m}$, in the context of Artificial Neural Networks, can be defined as the Weights and Bias of a typical ANN.

Therefore, this measure can be used, for example, to express how much the model output, $SoC_{m}$ = $f(X,\theta_{m})$, resembles the SoC output reference of the system. In this context, the correntropy can be applied to evaluate the performance of models obtained by the identification procedure. Under the mathematical definition of correntropy and its respective properties, it is concluded that the higher the value of the correntropy, the better is the quality of the model found, and consequently, the lower are the estimation errors of the SoC. From this point of view, the identification of the state of charge can be interpreted, therefore, as a procedure that aims to adjust the training parameters of the adaptive systems $\theta_{m}$ to maximize the correntropy or, in an analogous way, minimize the equation.

(5) $$J_{MCC}=\frac{1}{N}\sum_{k=1}^{N}\left(\frac{1}{\sigma \sqrt{2\pi }}-\frac{1}{\sigma \sqrt{2\pi }}exp^{-\frac{e_{k}^{2}}{2\sigma^{2}}}\right)$$

where: $e_{k}$ is the estimation error for the *k*-th training example and *N* is the number of samples collected from the system. The training algorithm will run in a sequential mode. Equation (6) defines the instantaneous expression of $J_{MCC}$ that will be used to update the weights and biases parameters after the presentation of each training pair.

(6) $$\varepsilon_{k}=\frac{1}{\sigma \sqrt{2\pi }}\left(1-exp^{-\frac{e_{k}^{2}}{2\sigma^{2}}}\right)$$

To perform the ANN learning procedure through the error backpropagation algorithm during your training process, you must calculate the partial derivatives of the chosen cost function in relation to the adjustable net parameters. These parameters can be updated from the delta rule principle, using (7) to (10).

(7) $$w_{ij(k+1)}=w_{ij(k)}-\eta \frac{\partial \varepsilon_{\left(k\right)}}{\partial w_{ij(k)}}$$

(8) $$w_{jd(k+1)}=w_{jd(k)}-\eta \frac{\partial \varepsilon_{\left(k\right)}}{\partial w_{jd(k)}}$$

(9) $$b_{j(k+1)}=b_{j(k)}-\eta \frac{\partial \varepsilon_{\left(k\right)}}{\partial b_{j(k)}}$$

(10) $$b_{d(k+1)}=b_{d(k)}-\eta \frac{\partial \varepsilon_{\left(k\right)}}{\partial b_{d(k)}}$$

where:

(11) 1≤i≤4

(12) 1≤j≤5

(13) d=1

where: *w* and *b* represents the adjustable parameters of the network through the learning procedure (weights and biases, respectively), η is the learning coefficient, *i* is the number of neurons of the input layer, *j* is the number of neurons of the hidden layer and *d* is the number of neurons of the output layer. Using this index notation, for example, the $w_{13}$ neuron connects I(k) to the third neuron of the hidden layer. Thus, the partial derivatives of ε(k) in relation to the adjustable parameters weights ($w_{ij}$ e $w_{jd}$) and biases ($b_{j}$ e $b_{d}$) are presented in Equations (14)–(17).

(14) $$\frac{\partial \varepsilon (k)}{w_{ij}\left(k\right)}=\frac{\partial \varepsilon (k)}{\partial e(k)}.\frac{\partial e(k)}{\partial w_{ij}\left(k\right)}=-\frac{1}{\sigma^{3}\sqrt{2\pi }}exp^{-\frac{e^{2}\left(k\right)}{2\sigma^{2}}}.e\left(k\right).x\left(k\right).w_{ij}\left(k\right)$$

(15) $$\frac{\partial \varepsilon (k)}{b_{j}\left(k\right)}=\frac{\partial \varepsilon (k)}{\partial e(k)}.\frac{\partial e(k)}{\partial b_{j}\left(k\right)}=-\frac{1}{\sigma^{3}\sqrt{2\pi }}exp^{-\frac{e^{2}\left(k\right)}{2\sigma^{2}}}.e\left(k\right).w_{ij}\left(k\right)$$

(16) $$\frac{\partial \varepsilon (k)}{w_{jd}\left(k\right)}=\frac{\partial \varepsilon (k)}{\partial e(k)}.\frac{\partial e(k)}{\partial w_{jd}\left(k\right)}=-\frac{1}{\sigma^{3}\sqrt{2\pi }}exp^{-\frac{e^{2}\left(k\right)}{2\sigma^{2}}}.e\left(k\right).a_{j}\left(k\right)$$

(17) $$\frac{\partial \varepsilon (k)}{b_{d}\left(k\right)}=\frac{\partial \varepsilon (k)}{\partial e(k)}.\frac{\partial e(k)}{\partial b_{d}\left(k\right)}=-\frac{1}{\sigma^{3}\sqrt{2\pi }}exp^{-\frac{e^{2}\left(k\right)}{2\sigma^{2}}}.e\left(k\right)$$

where: *x* represents the matrix with the input variables of the system, they are: the operating current, discharge voltage, and temperature, and σ represents the kernel width.

In a supervised training process, an epoch is counted at each presentation to the artificial neural network of all input-output pairs present in the training set. Thus, epoch after epoch, from Equations (14) to (17) in (7) to (10), the algorithm performs the adjustment of ANN parameters in order to maximize the correntropy through (5). It is important to comment that special attention must be given to the initialization of the Weights and Bias parameters. An inadequate initialization of these parameters can compromise the training, and consequently, the quality of the final model obtained. Proper choice increases the speed of training, resulting in rapid convergence.

## 6. Experimental Results

In this section, the performance of the adaptive control algorithm $RNA_{MCC}$ is evaluated through simulation tests, whose objective is to identify the state of charge. To achieve these objectives, two estimation scenarios were evaluated in this methodology, the first in the absence of outliers and noise, and the second in the presence of these contaminated samples. First, in both scenarios, three different *kernel* width values were analyzed: 1, 2, and 3. These values represent, respectively, low, medium, and high *kernel* widths for this case study. The simulation was performed using Matlab/Simulink on a Inter Core i7-7500U 2.70 GHz computer with 16.00 GB RAM. The time to perform each training plus the estimation time for the validation test lasted an average of 25 s.

Then, in order to evaluate the robustness of the proposed methodology over the classical methodology that uses an MSE-based cost function, a comparison between both methodologies is performed.

Before starting the presentation of results, some points need to be established. From now on, the notation $ANN_{MCC,AG}$ will be adopted to denominate the model obtained from the use of correntropia as a cost function of the error backpropagation algorithm and genetic algorithm for optimization of parameters η and σ and the notation $ANN_{MCC,ADAP}$ will be adopted to denote the estimation model obtained from the use of the adaptive *kernel* strategy.

### 6.1. Model Reference Definitions

In the training algorithm for the state of charge, shown in Figure 3, the SoC predictor corresponds to the estimation of the state of charge by Coulomb Counting, according to the following expression:

(18) $$SoC=SoC_{0}+\frac{100}{C_{n}}*\int_{t_{0}}^{t_{0}+\tau }Idt$$

where: $SoC_{0}$ represents the initial charge state equal to 1, $C_{n}$ corresponds to the nominal capacity of the battery equal to 6000 mAh, *I* represents the current flow in the battery, $t_{0}$ is the instant of time that the charge or discharge is initiated in the battery, and τ corresponds to the time interval between the beginning and end of the cycle. In the SoC predictor, only the *I* operating current and the relative time of the discharge cycle, coming from the database, is used.

### 6.2. Outliers and Noise Definitions

For the implementation of state of charge, several weights and analyzes are required regarding the battery model used. However, the model will still be subject to noise effects. This noise may be due to several factors such as: poor contact; use of poor quality sensors; A/D converters with low resolution; network noise that may propagate to the battery’s power source; during operation. In most of the articles reviewed previously the measurement noise is assumed to be Gaussian white noise. Thus, in one of the analyses of the designed model, the state of charge is contaminated by samples with normal distribution, zero average, and power equal to 0.01 W or −20 dB.

In order to simulate discrepant measurements, certain values are randomly inserted into the validation set, called as outliers. These outliers are formed by the followings expressions:

(19) $$SoC_{new,k}=1.2\cdot SoC\left(k\right)$$

(20) $$SoC_{new,k}=0.8\cdot SoC\left(K\right)$$

where SoC(k) is the original value of the SoC, without the presence of outliers or noises and $SoC_{new,k}$ is the outlier, calculated from an original SoC(k) sample. As you can see, there are two possible choices for SoC and during the validation procedure, either of the two Equations (19) and (20) can be used to form outliers.

### 6.3. Comparison of the Proposed Method for Different Kernel Widths and Optimization of the Genetic Algorithm in a Scenario without Noise and Outliers—Constant Current Discharge

In [39], a driving scenario for an electric vehicle is identified. In this driving scenario, the accelerator pedal signals are modeled. Also in [39], the charging and discharging characteristics of various types of batteries are examined according to the driving scenario presented. In this analysis, it can be seen that the behavior of the SoC is practically linear at different ranges of the driving scenario. To this end, the simulations in this subsection are done to identify a state of charge with a practically linear behavior.

In this subsection, estimation results will be presented in the context where the training samples are uncontaminated by noise and *outliers* with the estimation algorithm based on optimization of the parameters η and σ. In this estimation model based on genetic algorithm optimization, the identification structure is observed according to Figure 3.

For each combination of algorithm, *kernel* width and learning coefficient, 15 simulations of the training algorithm were performed. This was done in order to obtain the best model for each combination and to allow the calculation of some evaluation metrics between the data estimated by ANN and the theoretical data provided by the data set available in NASA’s online repository.

The implementation of the genetic algorithm for optimization of the ANN parameters has the following characteristics.

**Figure 5:**
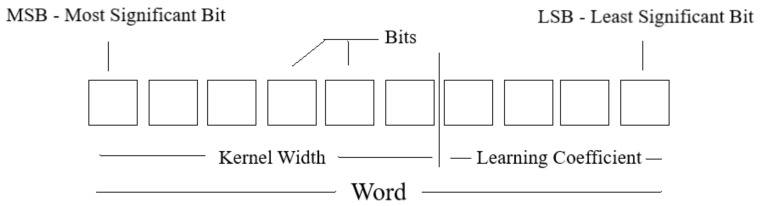
Individuals from the initial population.

1. *Population Generation*: Randomly generates a population of 250 individuals. Each individual contains information of a respective *kernel* width σ and a learning coefficient η. The individuals in the population are contained within the thresholds:
  (21) 0.2≤σ≤30
  (22) 0.2≤η≤15
  These limits were defined empirically. An extremely high value (η≫15) in the learning rate for this case study tends to create oscillations between the optimal learning point, so that the adjustment of the ANN weights does not converge to a satisfactory error. A small value (η≪1) makes the model training very slow, but the model converges to the optimal learning point. A weighting between model convergence time and the error in regime was established to determine the operating range of the learning rate η. Like the learning coefficient, the operating range of the kernel width is set with the goal of including a diverse population of kernels while maintaining population diversity [40]. This procedure prevents the algorithm from reaching a premature convergence condition [41]. The premature convergence in a genetic algorithm is when the algorithm reaches a minimum before it reaches the global optimum solution.
  After setting the thresholds of the population of individuals and randomly generating 250 individuals in binary form, each final individual contains information from both the learning coefficient η and the *kernel* width σ, as shown in Figure 5. This number of bits, as in Figure 5, does not represent the actual bit value used in this work. This figure is intended solely to help the reader visualize how a single individual contains information from two different quantities.
  The actual value of the number of bits for *kernel* width and learning coefficient, considering four decimal places of precision for both magnitudes and the thresholds exposed in Equations (21) and (22) is given by Equation:
  (23) $$N_{bits}=log_{2}^{Lim_{sup}-Lim_{inf}*10^{4}+1}$$
  where: $Lim_{sup}$ represents the upper threshold of the quantity being observed and analogously, $Lim_{inf}$ represents the lower threshold. Thus, the real value of bits for the *kernel* width is 19, considering that each individual, when in decimal form, presents 4 decimal places and 18 for the learning coefficient, presenting the same number of decimal places. Therefore, each final binary individual will have 37 bits in its representation, where the 19 most significant bits represent the width of the kernel and the 18 less significant bits represent the learning coefficient.
2. *Fitness*: After producing 250 individuals in their binary form, it is necessary to analyze the *fitness* of each individual, or analogously, to analyze the response the pair of quantities η and σ have on the artificial neural network. To perform this analysis, the width *kernel* and the learning coefficient σ are converted to decimal form and applied to a training on the neural network, where the error is given by expression (24):
  (24) $$Error=\frac{\sum_{k=1}^{N}e{\left(k\right)}^{2}}{N}$$
  where *N* represents the number of training samples and e(k) the best error obtained during the training process. Thus, the *fitness* of each individual is given by expression (25):
  (25) Fitness=100−Error
  After passing the *fitness* function, the individuals are sorted according to their *fitness* values. The best individual, that is, the one with the lowest error value, is chosen to integrate a new population. One way to make the individual directly proportional to its *fitness* value is to produce an expression according to the expression (25). In this way, the better the individual, the greater its *fitness*. The value 100 in this expression(25) was chosen so that the *fitness* value always presents positive values for this case study and thus can be used by the roulette wheel method of population classification.
  It is important to note that during the population training procedure, all individuals are subject to the same operating conditions, i.e., the training algorithm is initialized in the same way, with the same initial values of the adaptive parameters and with the same stopping criteria. This is done in order to evaluate only the effects that the learning coefficient and *kernel* width have on the estimation response, excluding unwanted effects such as adaptive parameter initialization.
3. *Crossover*: The crossover probability between two individuals in the population in the adopted strategy is 50%, where the information of the *kernel* width and learning coefficient, composing a single individual, were started in the genetic algorithm in binary form;
4. *Mutation*: The mutation probability adopted in the proposed method is 15% in order to maintain population diversity during the genetic convergence process.

**Figure 6:**
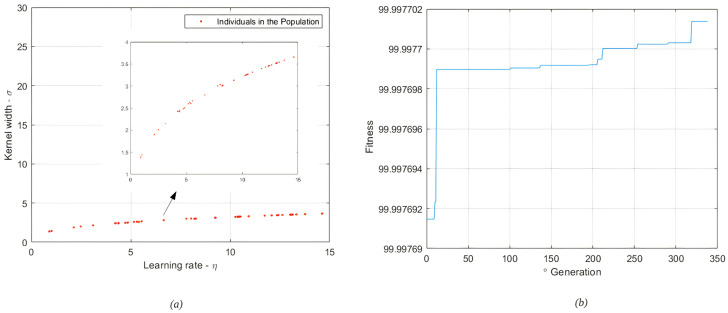
Population of individuals (**a**), Fitness of Generations (**b**). State of Charge—individuals of the population for training without the presence of noise and outliers—350${}^{\circ }$ generation.

Figure 6 illustrates the level of fitness of the population presented to the genetic algorithm. In Figure 6, it is observed that the genetic algorithm tries to privilege individuals with better aptitudes, thus trying to direct the search to regions of the search space where the optimal points are likely. The region with the best individuals, in this case, represents a search space limited by:

(26) 1≤σ≤4.5

(27) 1≤η≤15

Through the analysis of the evolution of the genetic algorithm, 330 generations was defined as the stop criterion. This number was defined using the idea of stagnation, that is, when no significant improvement of the population was observed after several consecutive generations, that is, when the average aptitude or the best individual did not improve anymore or when the aptitudes of the individuals in a population became very similar. The best individual in the population after this optimization process contains information from:

(28) $$\sigma_{best}=2.4203$$

(29) $$\eta_{best}=4.3807$$

When adjusting the parameters of the ANN models, with the exception of the $ANN_{MCC,AG}$, carried out by the error backpropagation algorithm, it was empirically verified that the best results were found when defining the learning coefficient η with the value equal to 1. A low learning rate tends to increase the model’s convergence time, since the adjustment of ANN parameters (Weights and Bias) is performed in a more subtle way. Rather than increasing times for training, the model’s error for such learning rates tends to decrease compared to high learning coefficients for the same operating conditions.

Although normally the learning curves practically stabilize around the 50th training generation, some good results were found with the execution of approximately 330 generation. For this reason, this last value was defined as the maximum number of seasons executed by the training algorithm.

**Figure 7:**
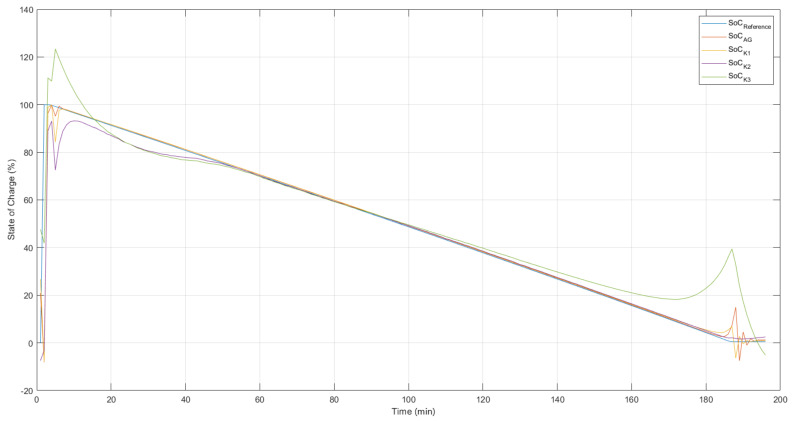
Validation of ANN models. State of Charge—training result without the presence of noise and outliers.

In Figure 7, the model responses are compared to the desired system output without the presence of noise and outliers. It is important to point out that during the training, the data set provided contained contaminated data and, even under these conditions, it is intended that, by using the cost function of the correntropy, it will be possible to obtain models capable of satisfactorily representing the dynamics of the state of charge for Lithium-ion 18650 batteries.

The best $ANN_{MCC,AG}$ model, whose answer is presented in the Figure 7, was obtained from the optimization of the *kernel* parameters and learning coefficient by the genetic algorithm.

Table 2 performs a performance comparison between the ANN models found when the experimental data was used in the identification. For each model configuration, the average of the validation MSE, the best MSE as well as the average of the validation MAPE are presented.

According to Table 2, the models obtained from the fixed kernel width equal to 3 did not perform well compared to the other models. When the width of *kernel* was fixed in a value considered too small, σ≤1, the $RNA_{MCC}$ models did not present a satisfactory result. The adjustment of the width of the *kernel* is directly related to the weights of the statistical moments of the Density and Probability Function of the error measured by this criterion. Thus, proposing small *kernel* width values means maximizing the amount of information contained in this distribution that will be used to update the ANN parameters. Setting a very small value for this parameter, however, creates, during training, a very restricted window of observation of the error distribution, inhibiting certain parameter adjustments that should be performed. When setting σ = 2 and σ = 3, there was a reduction in the performance of the $RNA_{MCC}$ models found. The best performing model corresponds to the $RNA_{MCC,AG}$ model with optimization of the η and σ parameters. This can be noted by both its average validation MSE and best validation MSE.

### 6.4. Comparison of the Proposed Method for Different Kernel Widths and Adaptive Strategy in a Scenario with Noise and Outliers—Constant Current Discharge

This section presents the results obtained in identifying the state of charge in Lithium-ion batteries, when the training and validation data used in the procedure are subject to the presence of white Gaussian noise and *outliers*.

The methodology used to obtain the results presented here is identical to the one used in the previous section. For each combination of algorithm, cost function and *kernel* width, 15 runs of the training algorithm were performed. Three different values of width of *kernel* (1, 2, and 3) were evaluated, as well as the adaptive adjustment of the kernel width. The value assigned to the learning coefficient η of the error backpropagation algorithm was equal to 1, and the number of training epochs was set to 500 epochs. The noise type used in the training procedure is white Gaussian noise and it is present in all samples. On the other hand, the outliers were inserted manually with discrepant values and are present only in certain samples.

In this section, during the training of the networks, an adaptive strategy is adopted to determine the kernel width. This strategy is based on Equation (30) [13].

(30) $$\sigma \left(k\right)=\frac{max|e_{i}\left(k\right)|}{2\sqrt{2}}$$

where: σ(k) corresponds to the kernel width value determined by the k-th training epoch and $e_{i}\left(k\right)$ corresponds to the estimation error generated by the i-th training example presented to the ANN.

The choice for an adaptive solution for the definition of the width of the kernel, in scenarios contaminated by noise and outliers, is due to the fact that the choice of a fixed value for this parameter that is efficient is usually not a simple task, especially when correntropy is used in nonlinear problems and in the presence of some stochastic characteristics, such as the identification of nonlinear systems and the supervised neural network training procedure.

**Figure 8:**
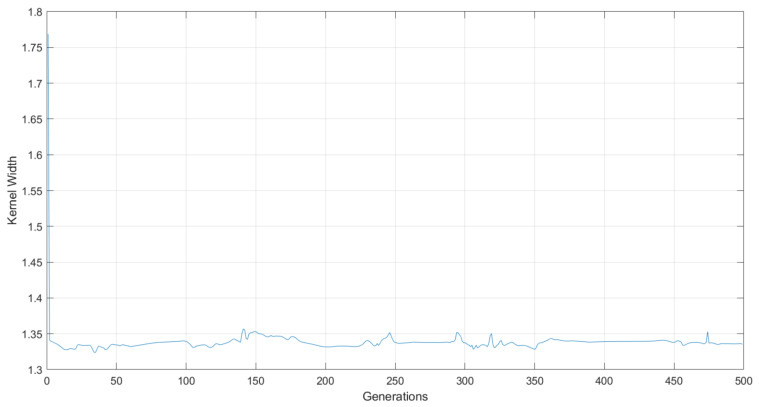
Kernel width variation during training.

The kernel width of the correntropy is initialized with a unitary value, presenting an increase in its value during the first epochs of the training algorithm. Then the value of this parameter starts oscillating, but with a tendency to decrease, until it stabilizes at a value close to 1.33, as show in Figure 8.

When adjusting the parameters of the ANN model, using the error back-propagation algorithm, it was empirically verified that the best results were obtained when setting the learning coefficient η to a value equal to 1. Although normally, the learning curves practically stabilize around the hundredth training epoch, some good results were found when running approximately 500 epochs. For this reason, this value was kept as the maximum number of epochs executed by the training algorithm. Thus:

(31) $$\sigma_{best}=1.33$$

(32) $$\eta_{best}=1.00$$

**Figure 9:**
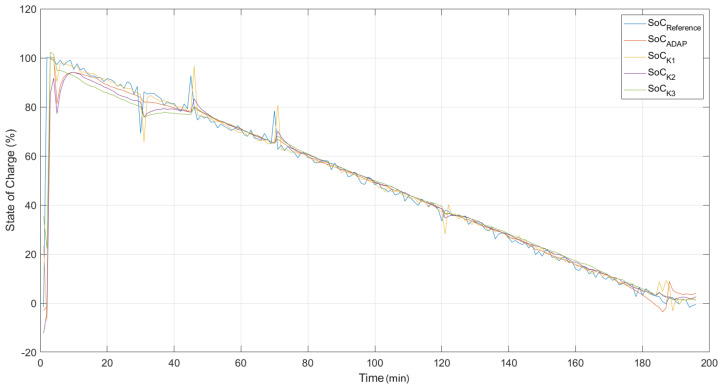
Validation of ANN models. State of Charge—training result with the presence of noise and outliers.

The best model $ANN_{MCC}$, whose response is presented in Figure 9, was obtained from the use of the adaptive *kernel* strategy. Analyzing this figure, it can be seen that this model presents the best performance among the models with various widths of *kernel*.

Table 3 presents a performance comparison between the $ANN_{MCC}$ models found when the experimental data applied to the SoC identification is subject to the presence of noise and *outliers*. Again, presented for each model configuration are the mean validation MSE, the variance of the validation MSE, and the best MSE found over 15 runs of the error backpropagation algorithm.

According to Table 3, the models obtained from the fixed width of the *kernel* equal to 1 did not present good performance when compared to the other models. The $ANN_{MCC,ADAP}$ model with adaptive *kernel* width showed the best result among the models with cost function in correntropy and presented more robustness in the presence of *outliers*, ignoring these discrepant measures in its identification process.

**Figure 10:**
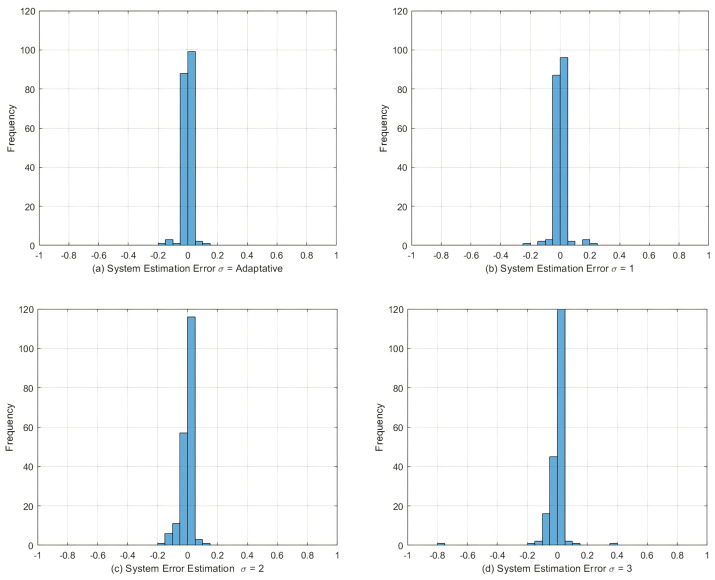
Histogram of the system estimation error. State of Charge—training result with the presence of noise and outliers.

The quantifications of the statistical moments of the histogram of Figure 10 are illustrated in Table 4, where the average is close to zero, as well as the standard deviation. The kurtosis parameter indicates how the tails of a distribution differ from the normal distribution. A distribution with a negative kurtosis value indicates that the distribution has lighter tails than the normal distribution, indicating a more “rounded” distribution, which is the case for all cases. The Skewness parameter indicates the asymmetry of the problem: as the data becomes more symmetric, its value tends to zero. For all kernel width variations, the Skewness value is close to zero.

### 6.5. Performance Comparison between MCC and MSE Criterion—Constant Current Discharge

This section presents the results obtained from the comparison between the models built from the classical MSE cost function and the correntropy based cost function, in a scenario contaminated with noise and outliers.

**Figure 11:**
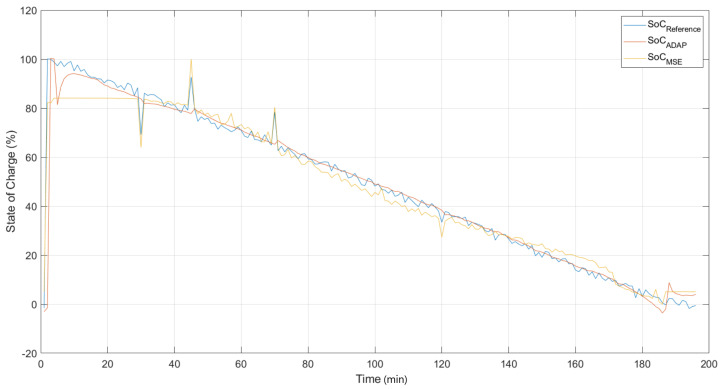
Validation of ANN models. State of Charge—training result with the presence of noise and outliers—Constant Current. Discharge.

In Figure 11, the responses of the $ANN_{MSE}$ and $ANN_{MCC,ADAP}$ models are compared with the desired output of the system with the presence of noise and *outliers*. It is important to point out that contaminated data was used during training and, even under these conditions, it is intended that, by using the correntropy cost function, it is possible to obtain models capable of satisfactorily representing the dynamics of the load state when it is under normal operating conditions. Comparing the $ANN_{MSE}$ and $ANN_{MCC,ADAP}$ models, it is observed that the second one presented a better performance. Although there was a percentage of *outliers* in the training and validation data sets, this did not provide a significant detrimental effect for the $ANN_{MCC,ADAP}$ model. This model was found by the error backpropagation algorithm, using adaptive *kernel* width and with a cost function defined by maximizing the correntropy.

Table 5 performs a performance comparison between the $ANN_{MSE}$ and $ANN_{MCC,ADAP}$ models found when the experimental data used for state of charge identification are subject to the presence of noise and outliers. Presented for each model configuration are the average validation MSE, the variance of the validation MSE, and the best MSE found over 15 runs of the training algorithm.

According to Figure 11 and Table 5, the correntropy is more robust than $ANN_{MSE}$ to the undesirable effects of large error values arising from outliers. This is because the MSE amplifies the contribution of points that are far from the average value of the error distribution, so if the error distributions contain outliers, are asymmetric or have a zero average, the criterion is not sufficiently robust.

The $ANN_{MCC}$ model with adaptive *kernel* width showed the best result among the models with cost function in correntropy and showed higher robustness and performance in the presence of outliers, ignoring these discrepant measures from its identification process, unlike the $ANN_{MSE}$ model. In nonlinear problems with high noise, in the presence of outliers, and non-Gaussian error distribution, the use of $ANN_{MSE}$ has limitations. This is due to the fact that MSE does not have any robustness to noise and is based only on the minimization of the error variance, not taking into account information of higher order statistical moments.

### 6.6. Performance Comparison between MCC and MSE Criterion—The Dynamic Stress Test

**Figure 12:**
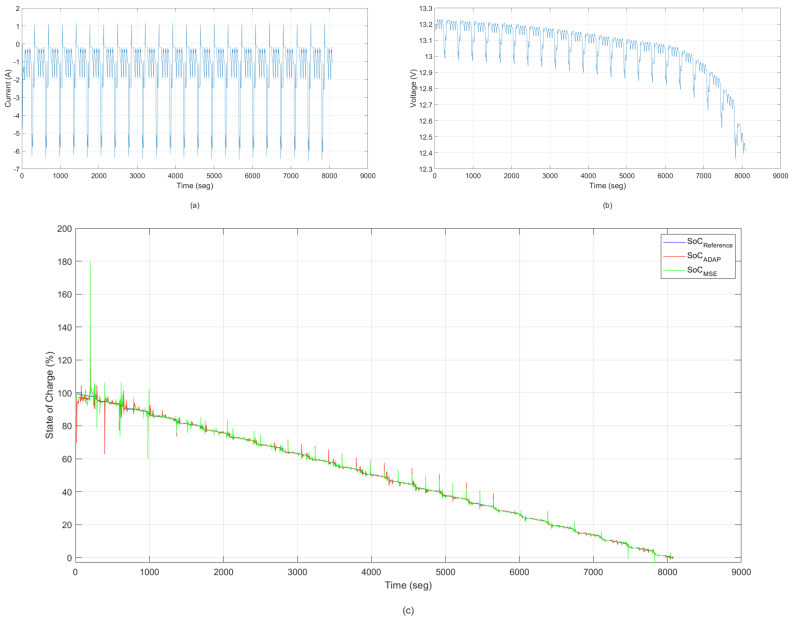
Validation of ANN models. State of Charge—training result without the presence of noise and outliers—Dynamic Stress Test: (**a**) current; (**b**) voltage for the DST cycle and (**c**) state of charge (SoC) estimation.

The DST test [42] is a widely used dynamic driving profile to evaluate the performance of the vehicle, and is also used to validate model accuracy or algorithm efficiency [43,44]. In the test, the battery goes through several DST cycles with the SoC operational range from 100% to 0%. The current and voltage profiles of the DST test are shown in Figure 12. To verify the robustness of the algorithms, the initials SoC are adjusted to 100%. This section is similar to the previous section, except that the validation test in this case is a test related to dynamic stresses of the battery. In this test, we used a dataset for Lí-ion batteries present in [42]. The shared dataset describes the behavior the Lí-ion (10 Ah) under the DST profile at room temperature in August 2016.

In Figure 12, the responses of the $ANN_{MSE}$ and $ANN_{ADAP}$ models are compared with the desired output of the system with the presence of noise and outliers. This validation test was constructed of extreme lithium-ion battery operating conditions (high temperature, relatively deteriorated battery capacity, pulsed current, and so on). The estimated model was built from the methodology based on an adaptive kernel-width strategy.

Table 6 shows a performance comparison between the $ANN_{MSE}$ and $ANN_{ADAP}$ models found when the experimental data used for state of charge identification are subject to the presence of noise and outliers. Presented for each model configuration are the average validation MSE, the variance of the validation MSE, and the best MSE found over 15 runs of the training algorithm.

Analyzing Figure 12 and Table 6, the model built with the correntropy-based cost function is more robust than the mean squared error-based model with respect to high error values. It can be seen that too large errors raise the value of the $J_{MCC}$ metric, which results in the error backpropagation algorithm changing the parameters (weights and bias) in order to reduce $J_{MCC}$. The way the backpropagation algorithm changes these parameters, either “intensely” or “smoothly” depends on the exact kernel width.

Another important point to comment on is that despite the training data being restricted to certain operational conditions, both in voltage levels as well as current and temperature levels, the model was able to estimate the state of charge quite satisfactorily. This response validates the model and demonstrates the robustness and adaptability of the neural network, even when subjected to operational conditions that are not present in the training data.

### 6.7. Performance Comparison between MCC and MSE Criterion—The Urban Dynamometer Driving Schedule

**Figure 13:**
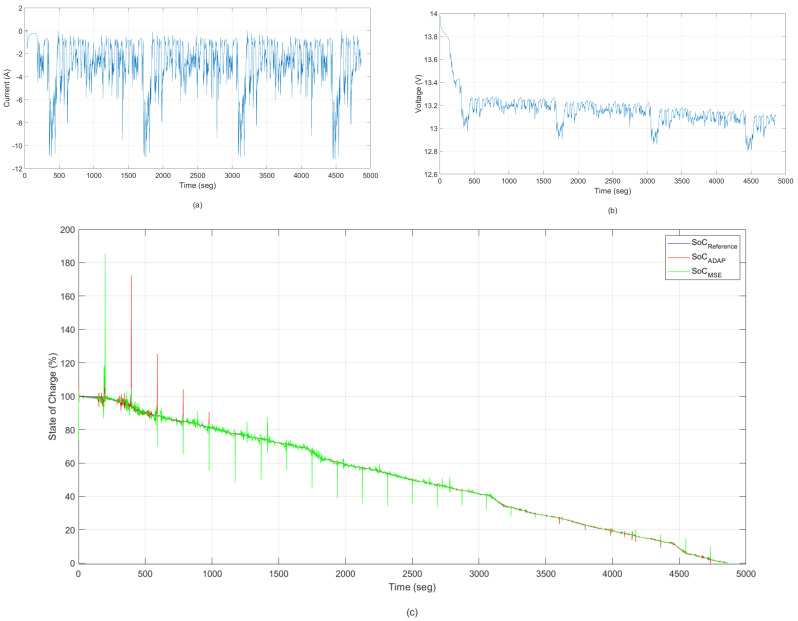
Validation of ANN models. State of Charge—training result without the presence of noise and outliers—The Urban Dynamometer Driving Schedule: (**a**) current; (**b**) voltage for the UDDS cycle and (**c**) state of charge (SoC) estimation.

The UDDS test [45] is another typical dynamic driving cycle to validate the usefulness of models and algorithms [43,46]. Similar to the DST, the battery goes through several UDDS cycles, and the SoC operational range is from 100% to 0%. The current and the voltage profiles on the test are shown in Figure 13, as well as the estimation of the SoC by both estimation methodologies.

It can be concluded from Figure 13 that $ANN_{MCC,ADAP}$ has the best performance in SoC estimation. To quantify the superiority of the proposed method, the statistical results are summarized in Table 7. For the model accuracy, the average mean square error between the estimated SoC by correntropy and the reference SoC model are 3.0349%. The model proposed by the classical methodology of the MSE has an average error of 4.0976%. Thus, the proposed methodology had a reduction of about 25% in relation to the classical methodology.

## 7. Conclusions

In this paper, an auto-regressive artificial neural network has been proposed to estimate the state of charge of Lithium-ion batteries. The methodology was developed following the concepts of Information Theory—more specifically, the definitions associated with Correntropy Maximization.

The main advantages of using the proposed identification method are listed:

- The use of the training algorithm based on the Correntropy Maximization Criterion allows outliers to be eliminated or ignored in the identification procedure;
- MCC allows higher order statistical moments to be taken into account during the parameter tuning procedure of the Artificial Neural Network.

In this paper, after writing in detail the architecture of the ANN used in this work, the modified equations of the error backpropagation algorithm resulting from the use of the correntropy as the basis of its cost function were presented, and it can be noted that two more adjustable parameters were included to the training algorithm: the width of the *kernel* of the correntropy and the learning coefficient.

In training the networks and consequently when obtaining ANN models, different fixed values for the width of the correntropy *kernel* were assigned and noise and different percentage values of *outliers* were inserted in the training and validation sets. Thus, it could be noted the importance of the proper adjustment of the *kernel* so that the modified algorithm can play its role satisfactorily.

In quantitative terms, the following results can be concluded:

- In a scenario without noise and outliers, the model based on correntropy with evolutionary strategy for kernel width optimization, presented the best result among several fixed kernel widths proposed in the methodology, containing a best error of $4.8\times 10^{-3}$ (see Figure 7). This implies that the proper choice of kernel width directly influences the estimation;
- In a scenario with noise and outliers, the model based on correntropy with adaptive strategy for kernel width optimization presented the best result among the several fixed kernel widths and in comparison with the classical method with cost function based on MSE. This model presented the best error of $2.5\times 10^{-3}$ (see Figure 9), and also showed more robustness to the presence of outliers in the reference signal;
- In both tests that simulate the actual behavior of a battery, either the DST test or the UDDS test, the correntropy-based methodology was able to satisfactorily approximate the reference SoC, keeping the performance metrics at values on the order of $10^{-2}$.

Therefore, the application of the correntropy measure in ANN training, when applied to the identification of nonlinear dynamic systems, can be considered an interesting alternative for estimation.

## Figures and Tables

**Table 1:** Electrical Characteristics Of The Battery 18.650.

Nominal capacity	6000 m Ah
Operating temperature	−5 ${}^{\circ }$C∼+60 ${}^{\circ }$C
Internal resistance	30 mΩ
Nominal voltage	3.7 V
Discharge cut-off voltage	2.5 V
Discharge current	1, 2 and 4 A

**Table 2:** Performance comparison of ANN models. State of Charge—Training result without the presence of noise or outliers.

Model and Training	Kernel Witdh	Average MSE of Validation	Average MAPE of Validation	Best MSE of Validation
$ANN_{MCC}$	Genetic Algorithm	5.00$\times 10^{-3}$	24.6$\times 10^{-3}$	4.80$\times 10^{-3}$
$ANN_{MCC}$	σ = 1	6.00 $\times 10^{-3}$	39.4 $\times 10^{-3}$	5.80$\times 10^{-3}$
$ANN_{MCC}$	σ = 2	11.45 $\times 10^{-3}$	44.3 $\times 10^{-3}$	10.94$\times 10^{-3}$
$ANN_{MCC}$	σ = 3	12.35 $\times 10^{-3}$	45.0 $\times 10^{-3}$	11.50$\times 10^{-3}$

**Table 3:** Performance comparison of ANN models. State of Charge—training result with the presence of noise or outliers.

Model and Training	Kernel Witdh	Average MSE of Validation	Average MAPE of Validation	Best MSE of Validation
$ANN_{MCC}$	Adaptative	2.5$\cdot 10^{-3}$	6.25 $\times 10^{-3}$	2.4$\times 10^{-3}$
$ANN_{MCC}$	σ = 1	7.4 $\times 10^{-3}$	51.5$\times 10^{-3}$	7.1$\times 10^{-3}$
$ANN_{MCC}$	σ = 2	6.9$\times 10^{-3}$	49.4$\times 10^{-3}$	6.7$\times 10^{-3}$
$ANN_{MCC}$	σ = 3	5.0 $\times 10^{-3}$	25.1$\times 10^{-3}$	4.8$\times 10^{-3}$

**Table 4:** Statistical measures of the estimation error when the system is subjected to the presence of noise and outliers.

	Erro de Estimação σ=Adaptative	Tracking Error σ=1	Tracking Error σ=2	Tracking Error σ=3
Average	0.4805	0.4842	0.4793	0.4844
Standard Deviation	0.2972	0.3000	0.2902	0.2845
Skewness	−0.0710	0.0113	−0.1272	−0.0863
Kurtosis	1.7386	1.7667	1.7385	1.7746

**Table 5:** Performance comparison of ANN models. State of Charge—training result with the presence of noise or outliers—Constant Current Discharge.

Model and Training	Kernel Witdh	Average MSE of Validation	Average MAPE of Validation	Best MSE of Validation
$ANN_{MSE}$	-	4.2 $\times 10^{-3}$	20.2 $\times 10^{-3}$	4.1$\times 10^{-3}$
$ANN_{MCC}$	Adaptative	2.5$\times 10^{-3}$	6.25 $\times 10^{-3}$	2.4$\times 10^{-3}$

**Table 6:** Performance comparison of ANN models. State of Charge—Training result without the presence of noise or outliers—Dynamic Stress Test.

Model and Training	Kernel Width	Average MSE of Validation	Average MAPE of Validation	Best MSE of Validation
$ANN_{MSE}$	-	2.5082 $\times 10^{-2}$	12,4 $\times 10^{-3}$	2.15$\times 10^{-2}$
$ANN_{MCC}$	Adaptive	2.2476$\times 10^{-2}$	9,6 $\times 10^{-3}$	1.99$\times 10^{-2}$

**Table 7:** Performance comparison of ANN models. State of Charge—training result without the presence of noise or outliers—The Urban Dynamometer Schedule.

Model and Training	Kernel Width	Average MSE of Validation	Average MAPE of Validation	Best MSE of Validation
$ANN_{MSE}$	-	4.0976 $\times 10^{-2}$	12.4 $\times 10^{-3}$	3.8467$\times 10^{-2}$
$ANN_{MCC}$	Adaptive	3.0349$\times 10^{-2}$	5.7 $\times 10^{-3}$	3.0016$\times 10^{-2}$

## Abbreviations

The following abbreviations are used in this manuscript:

ANN	Artificial Neural Network
ARX	Autoregressive-Exogenous
BMS	Battery Management System
CIM	Correntropy Induced Metric
EV	Electric Vehicles
HEV	Hybrid Electric Vehicles
MAPE	Mean Absolute Percentage Error
MCC	Maximum Correntropy Criterion
MSE	Mean Squared Error
NASA	National Aeronautics and Space Administration
PDF	Probability Distribution Function
SOC	State of Charge
SOH	State of Health
UFPB	Federal University of Paraiba
